# Expression of Concern: Construction of a binary S-scheme S-g-C_3_N_4_/Co-ZF heterojunction with enhanced spatial charge separation for sunlight-driven photocatalytic performance

**DOI:** 10.1039/d4ra90107j

**Published:** 2024-09-26

**Authors:** Ali Bahadur, Shahid Iqbal, Mohsin Javed, Syeda Saba Hassan, Sohail Nadeem, Ali Akbar, Rami M. Alzhrani, Murefah Mana Al-Anazy, Eslam B. Elkaeed, Nasser S. Awwad, Hala A. Ibrahium, Ayesha Mohyuddin

**Affiliations:** a Department of Chemistry, College of Science and Technology, Wenzhou-Kean University Wenzhou China; b Department of Chemistry, School of Natural Sciences (SNS), National University of Science and Technology (NUST) H-12 Islamabad Pakistan shahidiqbal.chem@sns.nust.edu.pk; c Department of Chemistry, School of Science, University of Management and Technology Lahore Pakistan; d Department of Physics, University of Agriculture Faisalabad (UAF) Faisalabad Punjab Pakistan; e Department of Pharmaceutics and Industrial Pharmacy, College of Pharmacy, Taif University P. O. Box 11099 Taif 21944 Saudi Arabia; f Department of Chemistry, College of Science, Princess Nourah bint Abdulrahman University P. O. Box 84428 Riyadh 11671 Saudi Arabia; g Department of Pharmaceutical Sciences, College of Pharmacy, AlMaarefa University Riyadh 13713 Saudi Arabia; h Chemistry Department, Faculty of Science, King Khalid University P. O. Box 9004 Abha 61413 Saudi Arabia; i Biology Department, Faculty of Science, King Khalid University P. O. Box 9004 Abha 61413 Saudi Arabia; j Department of Semi Pilot Plant, Nuclear Materials Authority P. O. Box 530 El Maadi Egypt

## Abstract

Expression of Concern for ‘Construction of a binary S-scheme S-g-C_3_N_4_/Co-ZF heterojunction with enhanced spatial charge separation for sunlight-driven photocatalytic performance’ by Ali Bahadur *et al.*, *RSC Adv.*, 2022, **12**, 23263–23273, https://doi.org/10.1039/D1RA08525E.

*RSC Advances* is publishing this expression of concern in order to alert readers that concerns have been raised over the integrity of the data published in this article. The authors have been contacted but have not provided the requested raw data. An expression of concern will continue to be associated with the article until a conclusive outcome is reached.

Laura Fisher

17th September 2024

Executive Editor, *RSC Advances*

